# Discrepancy between distribution of alpha-synuclein oligomers and Lewy-related pathology in Parkinson’s disease

**DOI:** 10.1186/s40478-022-01440-6

**Published:** 2022-09-06

**Authors:** Hiroaki Sekiya, Asato Tsuji, Yuki Hashimoto, Mariko Takata, Shunsuke Koga, Katsuya Nishida, Naonobu Futamura, Michi Kawamoto, Nobuo Kohara, Dennis W. Dickson, Hisatomo Kowa, Tatsushi Toda

**Affiliations:** 1 Department of Neuroscience, Mayo Clinic, 4500 San Pablo Road, Jacksonville, FL 32224 USA; 2 Division of Neurology, Kobe University Graduate School of Medicine, Kobe, Hyogo Japan; 3 Division of Molecular Brain Science, Kobe University Graduate School of Medicine, Kobe, Hyogo Japan; 4 Department of Neurology, National Hospital Organization Hyogo-Chuo Hospital, Sanda, Hyogo Japan; 5 Department of Neurology, Kobe City Medical Center General Hospital, Kobe, Hyogo Japan; 6 Department of Rehabilitation Science, Kobe University Graduate School of Health Sciences, Kobe, Hyogo Japan; 7 Department of Neurology, Graduate School of Medicine, The University of Tokyo, 7-3-1 Hongo, Bunkyo-ku, Tokyo, 113-8655 Japan

**Keywords:** Alpha-synuclein, Oligomers, Lewy bodies, Parkinson disease, Pathogenesis

## Abstract

The pathological hallmarks of Parkinson’s disease (PD) are α-synuclein (αSYN)-positive inclusions referred to as Lewy bodies and Lewy neurites, collectively referred to as Lewy-related pathology (LRP). LRP is thought to propagate in an ascending manner throughout the brain as the disease progresses. LRP is visible with histologic methods and is thought to represent a later stage of the disease process, while αSYN oligomers, which are not visible with routine histologic methods, are considered earlier. There is increasing evidence to suggest that αSYN oligomers may be more toxic than visible LRP. Detecting αSYN oligomers requires special techniques, and their distribution and association with clinical features are important research objectives. In this report, we describe the distribution of αSYN oligomers in multiple cortical and subcortical regions of PD using a proximity ligation assay (PLA). We observe widespread distribution of αSYN oligomers with PLA and more restricted distribution of LRP with αSYN immunohistochemistry. The distribution of αSYN oligomers differed from LRP in that αSYN oligomer burden was significantly greater in the neocortex, while LRP was greater in vulnerable subcortical regions, including the brainstem. We also found that cognitive impairment was associated with αSYN oligomers in the hippocampus. These results suggest that αSYN oligomers may be widely distributed in PD early in the disease process and that they may contribute to cognitive impairment in PD.

**Supplementary Information:**

The online version contains supplementary material available at 10.1186/s40478-022-01440-6.

## Introduction

Parkinson’s disease (PD) is a progressive neurodegenerative disorder characterized by bradykinesia, tremor, and rigidity [6, 35]. The pathological hallmark of PD is the Lewy body, a neuronal cytoplasmic inclusion found in selectively vulnerable neuronal populations [13]. The major structural component of Lewy bodies is α-synuclein (αSYN) [3, 40]. In addition to PD, dementia with Lewy bodies [39] and multiple system atrophy [2] are the most common α-synucleinopathies [12, 19, 25]. In addition to Lewy bodies, αSYN accumulation is also observed in glia and dystrophic neurites, the latter referred to as “Lewy neurites” [44]. Lewy bodies and Lewy neurites are collectively referred to as Lewy-related pathology (LRP) [18, 23]. LRP represents a later stage of aggregation of αSYN that is visible with histologic methods, while earlier stages of αSYN aggregation are not visible and have been referred to as αSYN oligomers [1]. Recent evidence suggests that αSYN oligomers may be more toxic than LRP [10, 11, 20, 22, 33, 46]. Braak et al. examined autopsy brains from 41 PD patients and 69 individuals without Parkinsonism and proposed a pathological staging of LRP in PD [9], based upon the hypothesis that αSYN propagation in human brains was not random, but followed a predictable pattern of selective vulnerability. While the general principles of the Braak PD staging scheme are valid, some patients do not fit the proposed staging scheme [4, 24, 34, 48]. One of the major correlates of the Braak staging hypothesis is that non-motor symptoms, such as rapid eye movement sleep behavior disorder, should precede motor symptoms due to earlier involvement of specific brainstem nuclei (e.g., pedunculopontine nucleus [17]) before the substantia nigra. On the other hand, it has been difficult to detect αSYN oligomers in pathological specimens, and the distribution of αSYN oligomers and their association with clinical features remain to be elucidated.

A novel technique to detect αSYN oligomers is with proximity ligation assay (PLA) [28, 36, 38]. This method has enabled detection of αSYN oligomers in pathological specimens and led to recognition that αSYN oligomers can be detected in brain regions previously not recognized to be vulnerable to LRP. In αSYN-PLA, two forms of oligonucleotides are attached to an epitope-blocking anti-αSYN antibody and serve as templates for the circulation of connector oligonucleotides by a ligase when αSYN oligomers exist and two molecules of αSYN are in close proximity (< 40 nm). In late-stage aggregates such as LRP, due to conformational changes, the antibodies do not bind close enough together and the reaction does not proceed [36]. The circularized DNA strands remain hybridized to the proximity probes; then, after the addition of DNA polymerase, the oligonucleotide arm of one of the PLA probes acts as a primer for a rolling-circle amplification (RCA) reaction using the ligated circle as a template, generating a concatemeric product that extends from the oligonucleotide arm of the PLA probe. The oligonucleotide of the second probe has three mismatched, exonuclease-resistant 2′ O-methyl RNA nucleotides at the 3′ end that prevent its use as a primer for RCA. RCA produces a randomly coiled, single-stranded product of up to 1000 complements of the DNA circle. Finally, oligonucleotides labeled with horseradish peroxidase hybridize to the RCA product and a visible signal is detected as a distinct red-brown precipitate after adding substrate. In our previous study, we noticed that αSYN oligomers are also widely distributed in PD brains, although less than in multiple system atrophy (MSA) [38], but the distribution of αSYN oligomers in PD brains has not previously been explored.

In the present study, we aimed to determine the distribution of αSYN oligomers using PLA, compare it with the distribution of LRP using α-synuclein immunohistochemistry in PD, and correlate oligomers with clinical features. We find that αSYN oligomers are more widespread than LRP and that αSYN oligomers in the hippocampus correlate with cognitive impairment.

## Materials and methods

### Brain samples and neuropathology

The present study included eight PD patients and five control subjects. Post-mortem brain samples from neuropathologically-confirmed cases of PD brain samples were obtained from the National Hospital Organization Hyogo-Chuo Hospital (Sanda, Hyogo, Japan), Kobe City Medical Center General Hospital (Kobe, Hyogo, Japan), and Kobe University Hospital (Kobe, Hyogo, Japan). We used five autopsy brains of subjects without parkinsonism and Lewy pathology from the National Hospital Organization Hyogo-Chuo Hospital as control. We reviewed medical records and collected clinical information of each patient. Written informed consent was obtained from the next of kin. This study was approved by the ethical committee of Kobe University Hospital.

We examined brain samples as previously reported in accordance with protocols in Research Resource Network Japan [38, 42]. Briefly, we stored portions of brain tissues were stored at − 80 °C and the rest of brain was fixed in 10% neutral buffered formalin. After fixation, the cerebrum was serially sliced in a coronal plane, the brainstem in an axial plane, and the cerebellum in a sagittal plane. Representative anatomical regions were embedded in paraffin. Serial 6-µm-thick sections were stained with hematoxylin and eosin (H&E). The following regions were examined: dorsal motor nucleus of the vagus, medullary raphe nuclei, locus coeruleus, substantia nigra, raphe nuclei of the midbrain, amygdala, entorhinal cortex, hippocampus, putamen, caudate, and neocortex (frontal, temporal, parietal, and occipital). Two continuous sections of each region were analyzed: one with phosphorylated-αSYN immunohistochemistry and one with αSYN-PLA.

### Clinical information and diagnosis of patients

All patients included in the present study were examined by multiple board-certificated neurologists between 2006 and 2017. We reviewed the medical records of each patient and collected the following clinical information: sex, age at onset, age at death, initial symptoms, visual hallucinations, and cognitive impairment. We considered a patient to have visual hallucinations if there were documented complaints from the patient or family members. A patient was considered to have cognitive impairment if a physician had diagnosed cognitive abnormalities, such as memory disturbance, disorientation, executive dysfunction, or stereotypic behavior [15]. We confirmed the pathological diagnosis of PD based on the presence of moderate to severe neuronal loss in the substantia nigra and the presence of LRP [14], which was assessed with H&E-stained sections and phosphorylated-αSYN immunohistochemistry of the midbrain. Control subjects were processed the same and had no neuronal loss or LRP in the substantia nigra.

### Immunohistochemistry

Immunohistochemistry for phosphorylated-αSYN was performed as described previously [38]. Paraffin-embedded brain sections were dewaxed in xylene, then rehydrated in a graded series of alcohol. Antigen retrieval was performed by microwave heating of slides for 15 min in pH6 citrate buffer. After blocking in 3% bovine serum albumin in phosphate-buffered saline at room temperature for 30 min, primary antibody for phosphorylated-αSYN (1:2000; mouse monoclonal, psyn#64, FUJIFILM Wako Pure Chemical Corporation, Osaka, Japan) was added and incubated at 4 °C overnight. Sections were washed in Tris-buffered saline (TBS) and were then incubated in hydrogen peroxide at room temperature for 30 min to inactivate endogenous peroxidase activity. After washing the sections, they were incubated with a biotin-conjugated secondary antibody (goat anti-mouse IgG) and then with avidin–biotin complex (VECTASTAIN Elite ABC Kit, Vector Laboratories, Burlingame, CA, USA). Phosphorylated-αSYN was visualized with 3,3′-diaminobenzidine-tetrahydrochloride-dihydrate and the sections were counterstained with hematoxylin. Samples were dehydrated in a graded series of alcohol and xylene before mounting with Permount mounting medium (Falma, Tokyo, Japan).

Braak neurofibrillary tangle (NFT) stage [7] and Thal amyloid phase [41] were assigned by thioflavin S fluorescent microscopy as previously described [37]. Immunohistochemistry for tau (AT8, mouse monoclonal, 1:2500, Thermo Fisher Scientific, Rockford, IL, USA) and phosphorylated transactive response DNA-binding protein of 43 kDa (pTDP-43) (pS409/410, mouse monoclonal, 1:5000, Cosmo Bio USA, Carlsbad, CA, USA) were performed on the hippocampus section of PD patients as previously described [31].

### αSYN-PLA staining

We conducted αSYN-PLA staining by using Duolink kits supplied by Sigma-Aldrich (St. Louis, MO, USA) to detect αSYN oligomers following the manufacturer instructions. We made both PLA probes with an αSYN antibody (mouse monoclonal, Syn211, Abcam, Cambridge, UK). We added 20 µg of Syn211 antibody to 2 µl of conjugation buffer and transferred the solution to a vial containing lyophilized oligonucleotides (plus or minus). Then, we incubated the solution at room temperature overnight. The conjugates were incubated with 2 µl of stop solution for 30 min at room temperature and suspended in 24 µl of storage solution. After dewaxing and hydrating the tissue sections as above, sections were incubated with hydrogen peroxide for 1 h at room temperature and subsequently heated in a microwave for 15 min in pH6 citrate buffer. Sections were blocked in 3% bovine serum albumin in phosphate-buffered saline at 37 °C for 1 h, followed by incubation with PLA probes diluted in PLA probe diluent (1:100) at 37 °C for 1 h, and then at 4 °C overnight. Sections were washed in TBS-T (TBS + 0.05% Tween 20) and then incubated with ligation solution and ligase at 37 °C for 1 h, washed in TBS-T and then incubated with amplification reagents and polymerase at 37 °C for 2.5 h. Finally, the sections were washed in TBS-T, incubated with detection solution at room temperature for 1 h, and finally incubated with substrate solution at room temperature for 20 min. Sections were counter-stained with hematoxylin and dehydrated in a graded series of alcohol and xylene before mounting with a bright-field mounting medium.

### Evaluation of LRP and αSYN oligomer burden

We assessed LRP (Lewy bodies and Lewy neurites) on phosphorylated-αSYN immunostained slides using a semi-quantitative scoring scheme at 20 × magnification. The severity of LRP was rated on a five-point scale: absent (0), slight (1+), mild (2+), moderate (3+), and severe (4+). A Braak PD stage was assigned to each case based on the distribution of LRP [8, 9].

**Fig. 1:**
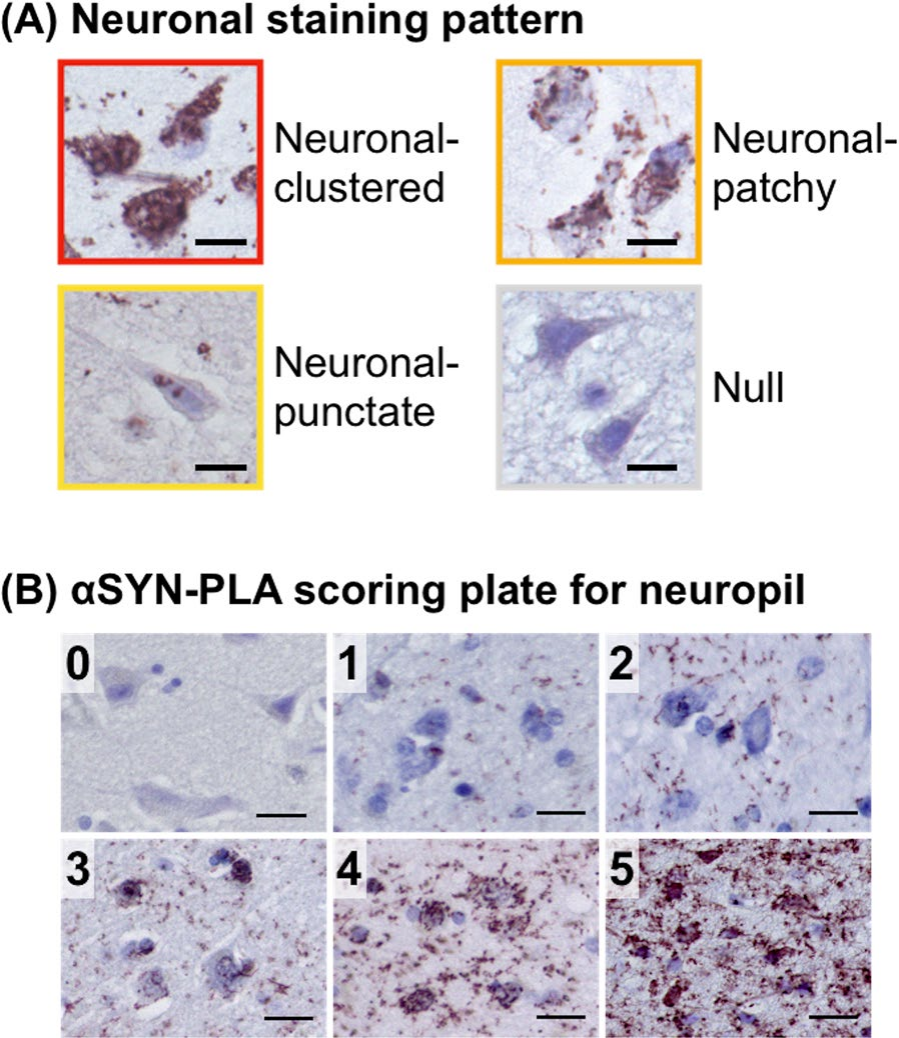
Evaluation of α-synuclein (αSYN) oligomers in neurons and neuropil. **A** Neuronal staining pattern of αSYN proximity ligation assay (αSYN-PLA) staining was classified into four patterns: neuronal-clustered, neuronal-patchy, neuronal-punctate, and null. Scale bars 10 µm. **B** αSYN-PLA severity in neuropil was scored from 0 (no signal) to 5 (highest) by a pre-made scoring plate. Scale bars 20 µm

We evaluated αSYN oligomer burden on αSYN-PLA slides as previously described [38]. Each stained slide was viewed at 20 × magnification and evaluated for the pattern and severity of neuronal and neuropil staining. Neuronal staining was classified based upon the staining pattern as follows: neuronal-clustered, neuronal-patchy, neuronal-punctate, and null (no signal detected) (Fig. 1A). Neuronal-clustered was characterized by αSYN-PLA signal throughout the neuronal perikarya; neuronal-patchy had patchy αSYN-PLA signal in the neurons; neuronal-punctate had dot-like αSYN-PLA signal. Neuropil staining was rated on a scale from zero (no signal) to five (highest signal) by using pre-made scoring plates (Fig. 1B). Because the cortical areas were large, we eliminated bias by evaluating αSYN-PLA neuropil scores in the three most affected microscopic fields and calculating the average scores in the cortex of superior frontal gyrus, superior temporal gyrus, superior parietal lobule, lingual gyrus, and parahippocampal gyrus. In other regions, such as the dorsal motor nucleus of the vagus, medullar raphe nuclei, locus coeruleus, substantia nigra, raphe nuclei of the midbrain, amygdala, hippocampus, putamen, and caudate, the region with the most signal was scored.

To compare the severity of αSYN oligomer burden with LRP, we made a combined severity score for αSYN oligomers on a five-point scale from the neuronal staining pattern and neuropil scores. We compared the score of LRP severity and combined severity of αSYN oligomer burden in each brain region. We also compared the mean pathology scores of each patient in the brainstem and neocortex to compare regional anatomical differences in the distribution of each pathology.

In addition to the scoring described above, we quantitatively measured the immunoreactive area of αSYN-PLA staining using the software ImageJ (National Institute of Health, Bethesda, MD, USA). The cortical images were used because they were large enough to fill all areas of 1720 × 1075 pixels at 20 × magnification. Three representative images of each cortex were obtained with the Aperio AT2 Slide Scanner (Leica Biosystems, Deer Park, IL, USA) at 20 × magnification. Quantification of immunostaining was performed using the IHC Image Analysis Toolbox plugin. After the training procedure with Nova Red color, the stained images were analyzed with color detection and converted to a 16-bit format (Additional file 1: Fig. S1A). Then, we measured the area of threshold pixels.

### Statistical analysis

We conducted all statistical analysis with GraphPad Prism (version 9.1.2, GraphPad Software, La Jolla, CA, USA). Unpaired t test was used to compare differences in age at death between PD and controls. We compared the LRP scores and combined αSYN oligomer score in each brain region by Wilcoxon matched-pairs signed-rank test. The combined αSYN oligomer scores and pathological burden scores in each brain region of PD and controls were compared with respect to clinical symptoms with unpaired t test (Welch’s correction was performed when the variance of the two groups was not equal). We calculated Spearman's rank correlation coefficient and examined whether there is a correlation between the duration of disease and the respective pathology scores. Statistical significance was defined by a p value < 0.05.

## Results

### Demographics and clinical characteristics

We summarize the demographic and clinical characteristics of each patient and control subject in Table 1. There was no significant difference in age at death between PD and control subjects (74 ± 10 years in PD patients and 64 ± 10 years in control subjects, respectively, P = 0.089). The median disease duration of PD patients was 15 ± 7 years. The initial symptoms of PD patients were tremor in four patients, gait difficulty in three patients, and writing disturbance in one patient. None of the control subjects had parkinsonism. Among eight PD patients, six had visual hallucinations and four developed cognitive impairment during their disease course.

**Table 1:** Demographic and clinical characteristics

ID	Sex	Age at Death (Y)	Disease duration (Y)	Initial symptom	Cognitive impairment	Visual hallucinations
PD1	M	79	9.9	Hand tremor	−	+
PD2	M	76	17.0	Hand tremor	−	+
PD3	M	88	7.9	Hand tremor	−	−
PD4	M	64	27.9	Writing difficulty	+	−
PD5	M	74	19.8	Gait difficulty	+	+
PD6	M	59	13.9	Hand tremor	+	+
PD7	F	68	11.8	Gait difficulty	−	+
PD8	F	85	7.8	Gait difficulty	+	+
C1	F	55	NA	NA	−	−
C2	F	60	NA	NA	−	−
C3	M	60	NA	NA	−	−
C4	M	63	NA	NA	−	−
C5	F	80	NA	NA	−	−

*Y*, years; *ND*, no data; *NA*, not assessed

#### Pathologic features

The pathologic features of all cases are summarized in Table 2. Among PD patients, two cases were Braak NFT stage 0, three were stage I, and three were stage III. Thal amyloid phase was 0 in four patients, 1 in one patient, 2 in two patients, and 3 in one patient. One patient had TDP-43 pathology in the hippocampus.

**Table 2:** Pathological characteristics

ID	PMI (hr)	Braak NFT stage	Thal amyloid phase	Braak PD stage	TDP-43 in hippocampus
PD1	1.9	III	0	3	−
PD2	2.0	0	0	4	−
PD3	3.4	I	1	4	−
PD4	2.5	I	0	4	−
PD5	16.0	III	0	5	+
PD6	4.2	I	3	5	−
PD7	3.3	0	2	6	−
PD8	10.2	III	2	6	−
C1	1.5	NA	NA	NA	NA
C2	3.5	NA	NA	NA	NA
C3	7.7	NA	NA	NA	NA
C4	5.5	NA	NA	NA	NA
C5	ND	NA	NA	NA	NA

*PMI*, post-mortal interval; *hr*, hours; *NFT*, neurofibrillary tangle; *PD*, Parkinson’s disease; *TDP-43*, transactive response DNA binding protein of 43 kDa; *ND*, no data; *NA*, not assessed

### Distribution of αSYN oligomers and evaluation of αSYN oligomer severity

**Fig. 2:**
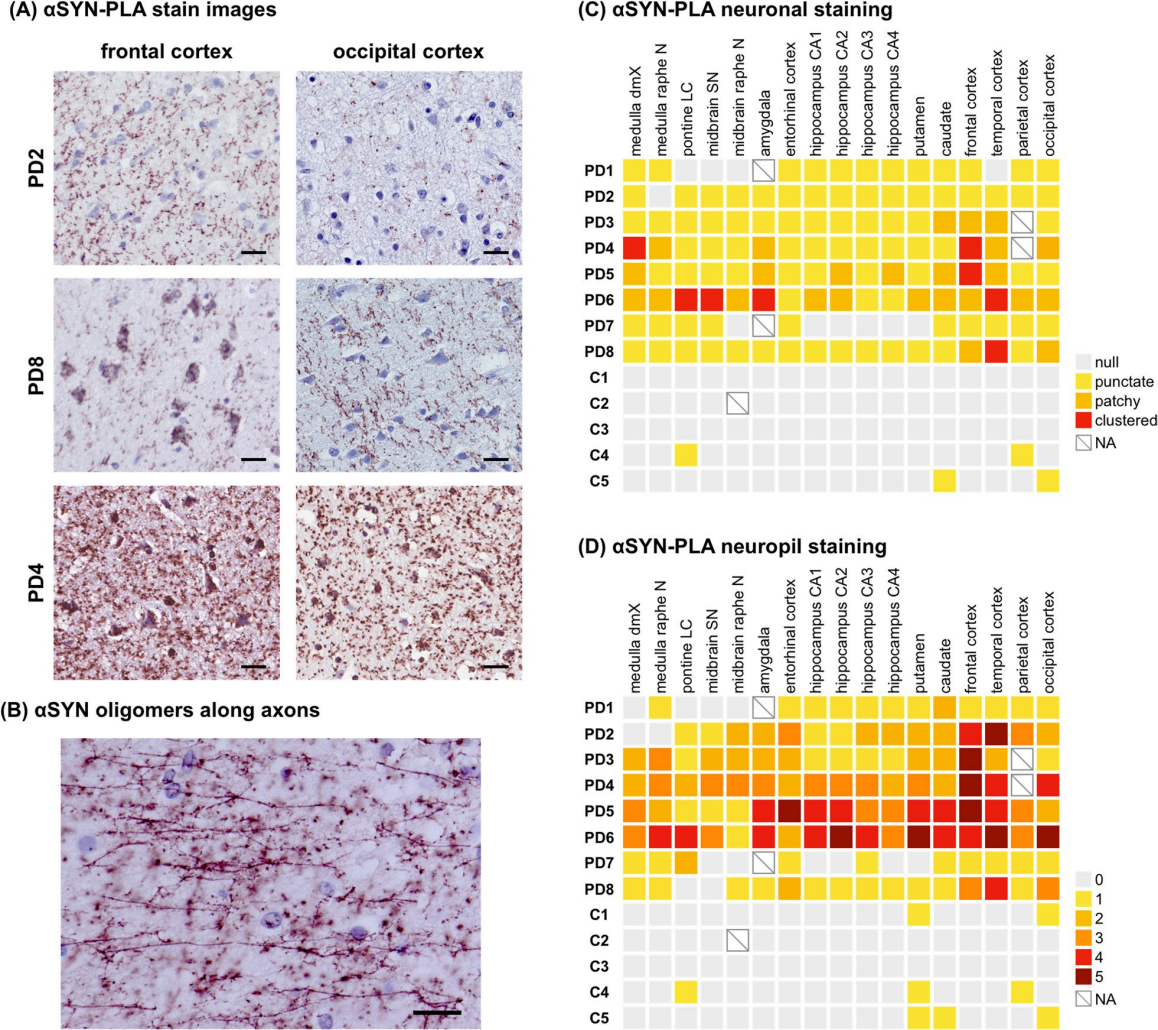
α-synuclein (αSYN) oligomers detected by αSYN proximity ligation assay (αSYN-PLA) staining. **A** Representative images of αSYN-PLA staining from three PD patients in the frontal cortex and occipital cortex. Scale bars 20 µm. **B** Linear staining along axons in the temporal cortex of PD2 detected by αSYN-PLA staining. Scale bar 20 µm. **C** Neuronal staining patterns of αSYN-PLA staining were classified into neuronal-clustered, neuronal-patchy, neuronal-punctate, and null. Each staining pattern in each brain region was shown in red, orange, yellow, and gray, respectively. **D** Severity of αSYN-PLA staining in neuropil was evaluated by the scoring plate. Each score was shown in red-brown, red, dark orange, light orange, yellow, and gray. *dmX*, dorsal motor nucleus of the vagus; *N*, nucleus; *LC*, locus coeruleus; *SN*, substantia nigra; *CA*, cornu ammonis; *NA*, not assessed

We examined the distribution and pathological burden of αSYN oligomers in neurons and neuropil on αSYN-PLA stained slides (Fig. 1). We illustrate representative αSYN-PLA staining in Fig. 2A. In the neocortex of PD2, αSYN oligomers were more prominent in neuropil than neuronal perikarya. A similar trend was observed in the neocortex of PD4, but we observed abundant αSYN oligomers in both neurons and neuropil. PD8 showed an intermediate degree of staining in neurons and neuropil between PD2 and PD4 with more prominent signals in neuropil. A linear staining pattern of αSYN-PLA, consistent with accumulation of synuclein oligomers in axons, was noted in some PD patients (Fig. 2B).

We assessed the pattern of αSYN oligomers in neurons. Neuronal staining was evaluated as neuronal-clustered, neuronal-patchy, neuronal-punctate, or null (Fig. 1A). The results are summarized in Fig. 2C. The neuronal-clustered pattern was observed in the brainstem of two patients (PD4 and PD6). The neuronal-clustered pattern was found in the neocortex of four patients (PD4, PD5, PD6, and PD8). The amount of αSYN-PLA neuropil staining was rated on a 0 to 5 scale by comparing images to pre-made scoring plates (Figs. 1B, 2D). We confirmed the correlation between the neuropil staining score and the stained area of αSYN-PLA staining (r = 0.8222, P < 0.0001, Additional file 1: Fig. S1B). There was a strong correlation between the severity of neuropil staining and the severity of neuronal staining in each brain region (r = 0.7010, P < 0.0001). The severity of neuropil staining tended to be higher in brain regions with the most neuronal staining. When we compared the severity of neuronal and neuropil staining in each region, neuropil staining was greater than neuronal staining in many regions. The severity of neuronal staining was greater than neuropil staining in only 3 of 132 regions examined (17 regions in 8 PD cases, excluding 4 missing).

**Fig. 3:**
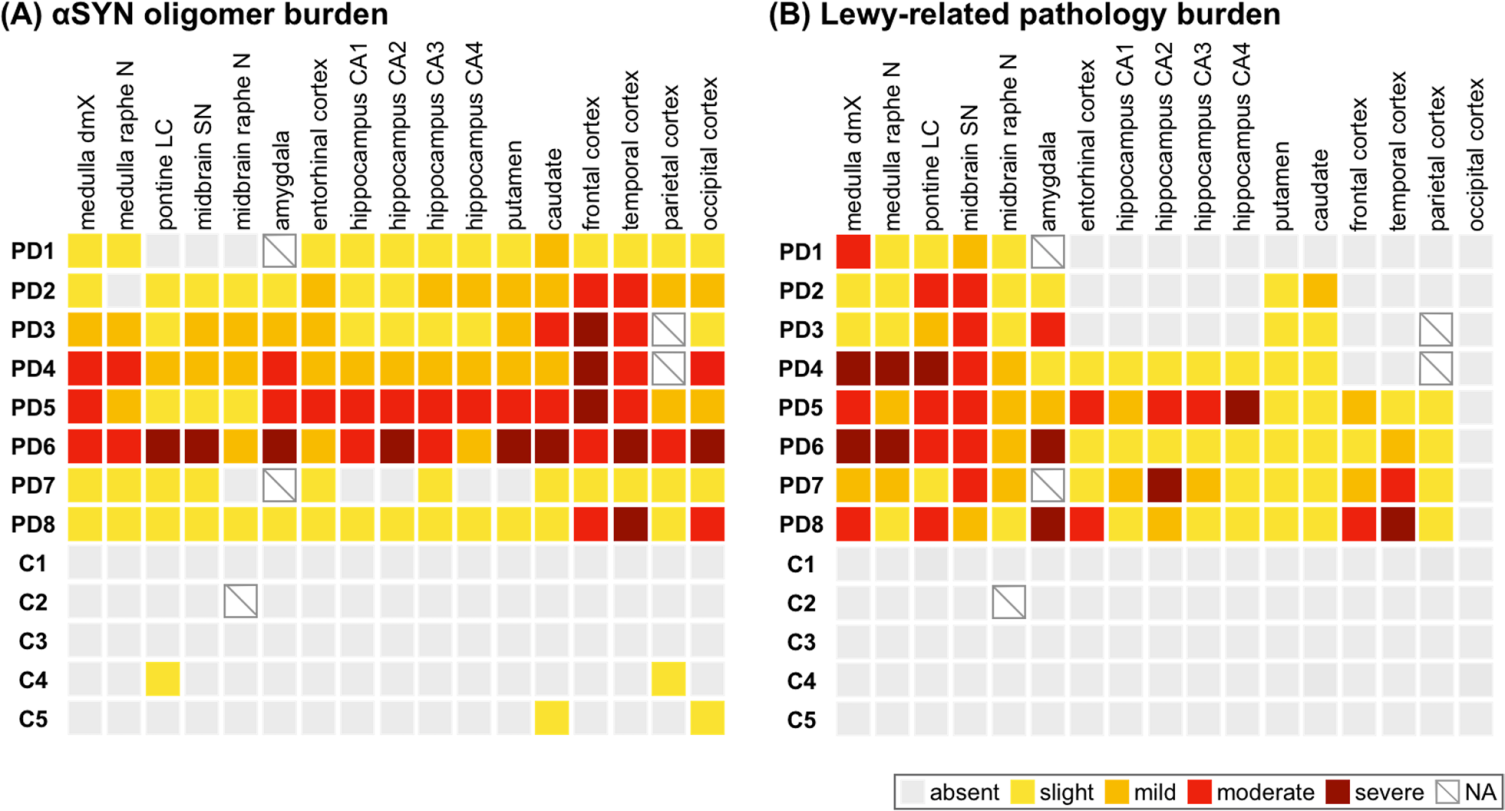
Comparison between α-synuclein (αSYN) oligomer burden and Lewy-related pathology (LRP) burden. **A** The overall severity of αSYN oligomer burden on a five-point scale in each brain region. **B** The severity of LRP burden on a five-point scale in each brain region. Each pathological severity (severe, moderate, mild, slight, and null) was shown in brown, red, orange, yellow, and gray, respectively. *dmX*, dorsal motor nucleus of the vagus; *N*, nucleus; *LC*, locus coeruleus; *SN*, substantia nigra; *CA*, cornu ammonis; *NA*, not assessed

We assessed the overall burden of αSYN oligomers on a five-point scale by combining the staining of neurons and neuropil. The results are shown in Fig. 3A. αSYN oligomers were more prominent in the neocortex than in the brainstem. In the limbic system, one patient had severe, one had moderate, two had mild, and four had mild burden of aSYN oligomers in the hippocampus. Six out of eight patients had moderate to severe burden of αSYN oligomers in the neocortex. We compared the severity of αSYN oligomers in each brain region between PD patients and control subjects. The αSYN oligomer severity score and stained area of PD patients were significantly greater than those of controls, respectively (Additional file 1: Figs. S2 and S3).

### Severity of LRP

We analyzed the severity of LRP (Lewy bodies and Lewy neurites) in each region with phosphorylated-αSYN immunohistochemistry and evaluated the severity on a five-point scale (0—no staining, 1—slight, 2—mild, 3—moderate, and 4—severe). We assessed Braak PD staging based on the distribution of LRP [8, 9]. There was one patient with Braak stage 3, three patients with Braak stage 4, two patients with Braak stage 5, and two patients with Braak stage 6. All PD patients had LRP in the brainstem, including the dorsal motor nucleus of the vagus, locus coeruleus, and substantia nigra. No PD patient had LRP in the occipital cortex. The severity of LRP in each brain region is summarized in Fig. 3B. Most of the cases had moderate to severe LRP in the brainstem. With respect to LRP in the limbic system, two patients had severe pathology, one patient had moderate pathology, one patient had mild pathology, and two patients had minimal pathology in the amygdala. Four out of eight patients had no LRP in the neocortex.

### Comparison of LRP and αSYN oligomer burden

**Fig. 4:**
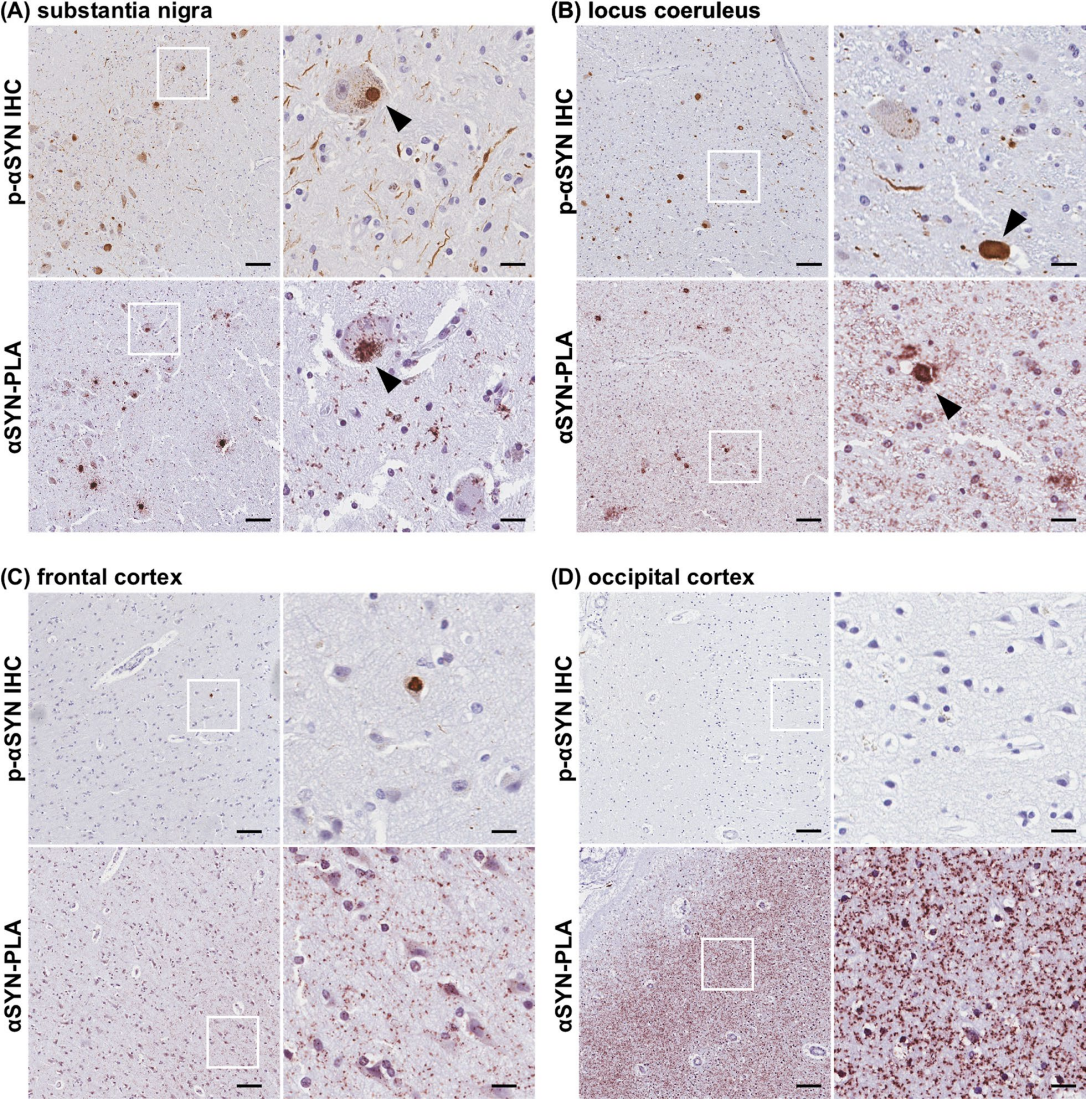
Comparative images of phosphorylated α-synuclein (αSYN) immunostaining and αSYN proximity ligation assay (αSYN-PLA) staining. Upper images are phosphorylated-αSYN immunostaining and lower images are αSYN-PLA staining. The left images are low-magnification images and the right images are high-magnification images of the area circled by the white squares. **A** Substantia nigra, **B** Locus coeruleus, **C** Frontal cortex, and **D** Occipital cortex. Scale bars 100 µm in low-magnification images, 20 µm in high-magnification images. *p-αSYN*, phosphorylated α-synuclein; *IHC*, immunohistochemistry; *PLA*, proximity ligation assay

We compared the severity of LRP and αSYN oligomers in the respective brain regions of PD patients. The representative comparative images of phosphorylated-αSYN immunostaining and αSYN-PLA staining are shown in Fig. 4. Lewy bodies were present in the substantia nigra, locus coeruleus, and frontal cortex, and αSYN oligomers were observed in the same regions. We observed αSYN oligomers at the periphery of some Lewy bodies in the substantia nigra and locus coeruleus (Fig. 4A, B, and Additional file 1: Fig. S4). αSYN oligomers in neuropil were more prominent than Lewy neurites (Fig. 4C). Although no LRP was detected, abundant αSYN oligomers were detected in the occipital cortex (Fig. 4D).

**Fig. 5:**
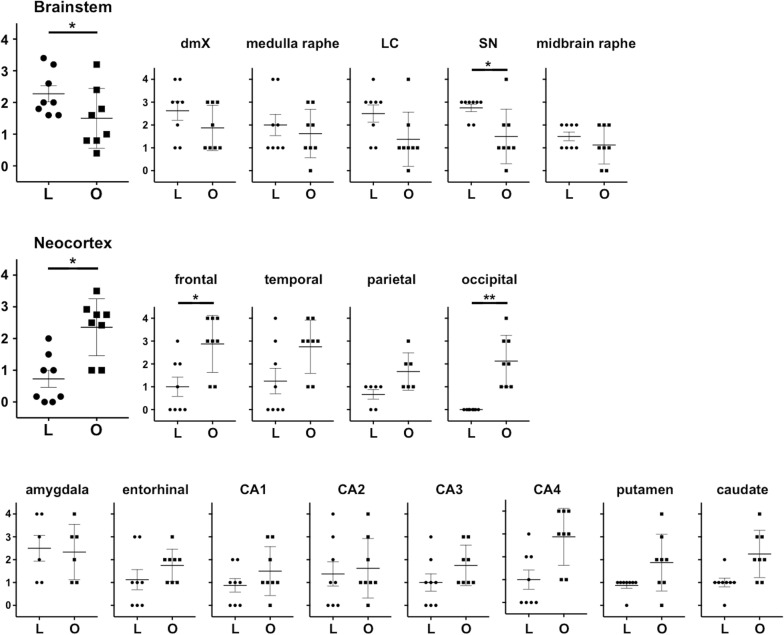
Each pathological severity score. *L,* Lewy-related pathology burden; *O*, α-synuclein oligomer burden; *dmX*, dorsal motor nucleus of the vagus; *LC*, locus coeruleus; *SN*, substantia nigra; *frontal,* frontal cortex; *temporal,* temporal cortex; *parietal,* parietal cortex; *occipital,* occipital cortex; *entorhinal,* entorhinal cortex; *CA*, cornu ammonis; *P < 0.05; **P < 0.01

We analyzed differences in the distribution of αSYN oligomers and LRP graphically (Fig. 5). LRP was significantly higher than αSYN oligomer score in the brainstem (P = 0.031), whereas the αSYN oligomer score was significantly higher than LRP in the neocortex (P = 0.016). Comparison of pathological severity in each brain revealed that αSYN oligomer score was significantly higher than the LRP in the frontal cortex (P = 0.047) and occipital cortex (P = 0.0078). The LRP score was significantly higher than αSYN oligomer score in the substantia nigra (P = 0.039).

### Clinical characteristics and each pathological severity

**Fig. 6:**
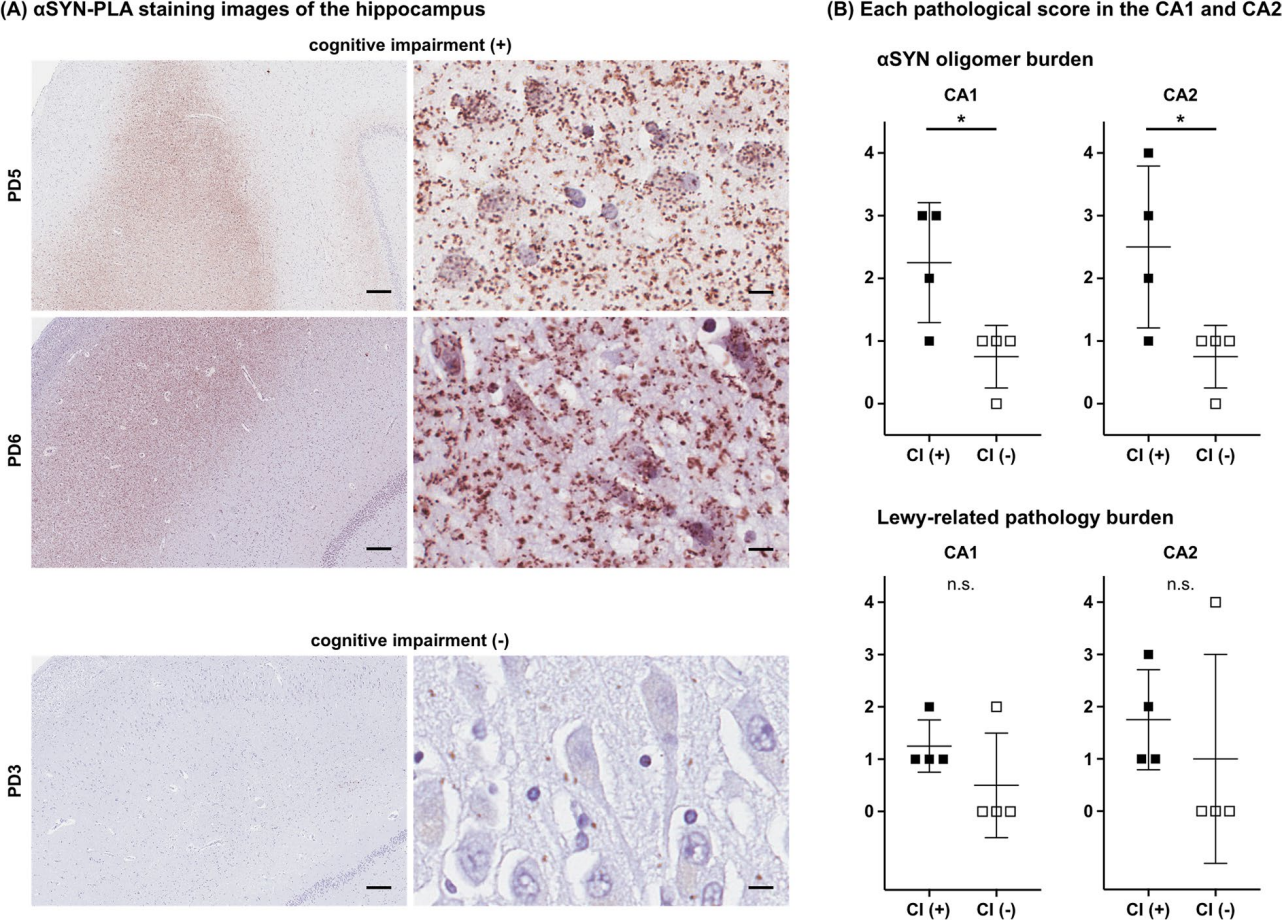
α-synuclein (αSYN) oligomers in the hippocampus in patients with or without cognitive impairment. **A** αSYN-PLA staining images of PD patients with or without cognitive impairment. The upper two images are from patients with cognitive impairment and the lower image is from a patient without cognitive impairment. The left images are low-magnification images of the hippocampus, including the granule cell layer and CA2. The right images are high-magnification images of the pyramidal cells in the CA2. **B** Each pathological score in the CA1 and CA2 between patients with and without cognitive impairment. Black squares represent the scores of patients with cognitive impairment; white squares represent the scores of patients without cognitive impairment. Scale bars 100 µm in low-magnification images, 10 µm in high-magnification images. *CA,* cornus ammonis; *CI*, cognitive impairment; *P < 0.05

Finally, we examined correlations between the severity of LRP and αSYN oligomers with clinical features. PD patients who had cognitive impairment had significantly higher αSYN oligomer scores in CA1 and CA2 of the hippocampus than those who did not have cognitive impairment (P = 0.032 and P = 0.045; Fig. 6). On the other hand, there was no significant difference in the severity of LRP score in the hippocampus between those with and without cognitive impairment. Braak NFT stage and Thal amyloid phase did not differ significantly between PD patients with and without cognitive impairment (P = 0.32 and P = 0.59). We found no significant correlation between the presence of visual hallucinations and either LRP or αSYN oligomer in the amygdala and occipital cortex. There was no significant correlation between disease duration and brainstem LRP severity (r = 0.65, P = 0.089), neocortical LRP score (r = -0.28, P = 0.50), brainstem αSYN oligomer severity score (r = 0.31, P = 0.45) or neocortical αSYN oligomer score (r = 0.45, P = 0.27).

## Discussion

Here we report a distribution of α-synuclein oligomers in PD using a PLA. In the present study, we found a widespread distribution of αSYN oligomers in PD brains and discordance between the distribution of αSYN oligomers and LRP. We also found that PD patients with cognitive impairment had more severe αSYN oligomers in the hippocampus.

Our results demonstrate that the distribution of αSYN oligomers was more widespread than that of LRP. Half of the patients in the present study had no LRP in the neocortex, but at least some αSYN oligomer was detected in the neocortex of all patients. Therefore, αSYN oligomers may be widespread even early in the disease stage. Accumulation of αSYN oligomers is rarely if ever observed in control subjects, therefore, αSYN oligomers detected with αSYN-PLA reflects genuine pathological findings related to PD. This is supported by previous in vitro and in vivo studies. αSYN-PLA staining has detected αSYN oligomers in two different methods (bimolecular fluorescence complication and FK506 binding protein-FK506 rapamycin binding-rapamycin model) [36]. Another study successfully detected αSYN oligomers in transgenic mice (Thy1-h-αSYN*A30P) with αSYN-PLA similar to those employed in the present study. Of note, no signal was observed in the non-transgenic control mice [5].

Regarding the association between clinical features and α-synuclein pathology, in the present study, more αSYN oligomers were found in the hippocampus of patients with cognitive impairment. This association was not observed with respect to LRP. There were also no differences in Braak NFT stage or Thal amyloid phase between patients with and without cognitive impairment. One patient with cognitive impairment had TDP-43 pathology in the hippocampus, which might have contributed to cognitive impairment [32, 43]. Mounting evidence suggests that αSYN oligomers are more toxic than LRP [11, 22, 46]. A recent study also reported MSA patients with cognitive impairment had more αSYN oligomers [30]. Therefore, cognitive impairment observed in the present study may be associated with αSYN oligomers.

**Fig. 7:**
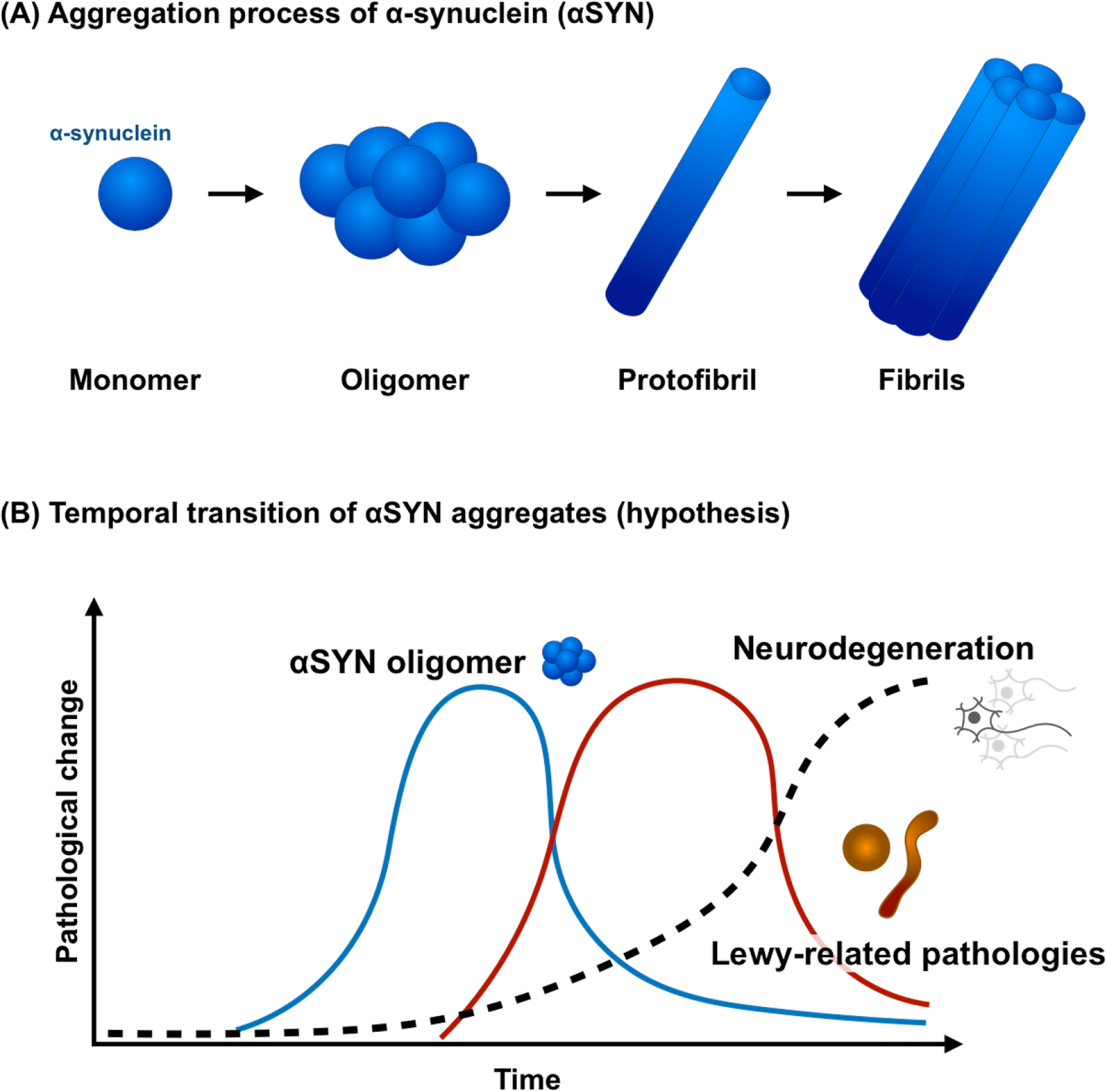
α-synuclein (αSYN) aggregation process and temporal accumulation of each aggregate (hypothesis). **A** Monomeric αSYN molecules assemble to form αSYN oligomers. As aggregation proceeds, they develop protofibrils and eventually fibrils. **B** A hypothetical accumulation process of αSYN aggregates in Parkinson's disease could include the following. αSYN oligomers fill first in neuropil and then accumulate in neurons. As Lewy-related pathology forms and accumulates, synuclein oligomers diminish, and Lewy-related pathology becomes predominant. Furthermore, as the disease progresses to the terminal stage and neuronal loss becomes more severe, the Lewy-related pathology also decreases

We also observed differences in the distribution between αSYN oligomers and LRP, with prominent αSYN oligomer burden in the neocortex and prominent LRP in the brainstem. The reason for this distribution discrepancy could be attributed to regional differences in neuronal aggregation of αSYN. In this study, all patients had neuropathologic and clinical diagnosis of PD, with motor symptoms prior to onset of any cognitive deficits. Accordingly, αSYN pathology is likely to have first appeared in the brainstem rather than the cortex. Based upon studies of αSYN aggregation, it is likely that αSYN oligomers precede formation of LRP [47]. We speculate that the reason for differences in distribution between αSYN oligomers and LRP is that αSYN oligomers are the substrate for LRP and that as LRP increases there is a shift in the pool of oligomers to fully formed fibrils in LRP. Since the neocortex is affected later in the disease of PD, relatively more αSYN oligomers than LRP are observed in the neocortex (Fig. 7).

The present study suggests that αSYN oligomers may propagate through axons and accumulate in neuropil before they accumulate in perikarya of neurons. We observed more αSYN oligomers in the neuropil than in neurons in most brain regions. These results suggest that αSYN oligomers appear first in neuronal processes in the neuropil, before that mature into LRP in perikarya. Moreover, we found a linear staining pattern of αSYN oligomers in the neocortex of PD patients that appeared to be in axons. This suggests that αSYN oligomers may propagate through axons.

Our results may be useful in considering the suitable targets for anti-αSYN therapy. In the Braak hypothesis, it has been thought that neurodegeneration progresses through the propagation of Lewy pathology [9]. This propagation concept was supported by the presence of Lewy bodies in neurons of fetal graft has suggested that pathological αSYN propagates [26, 27, 29]. A treatment strategy to stop the propagation of pathological synuclein has been considered [45]; however, anti-αSYN therapy has failed to demonstrate therapeutic efficacy [16]. The reason for the failure could be the widespread distribution of αSYN oligomers in earlier pathological stages of the disease. The successful treatment of PD patients with the anti-αSYN approach requires including very early PD or even those in a preclinical phase of the disease [21].

We recognize the relatively small sample size as a limitation. The implications of αSYN oligomers in the hippocampus on cognitive impairment warrant further research. Another potential limitation of the current study lies in the inherent nature of retrospective autopsy studies. Patients did not undergo the prospective clinical evaluations. Therefore, it remains possible that some of the clinical features may be underestimated. Nevertheless, the present study sheds new light on the neuropathology of PD from the perspective of αSYN oligomers.

## Conclusion

We examined the distribution of αSYN oligomers in formalin-fixed paraffin-embedded brain samples from patients with PD. The distribution of αSYN oligomers was more widespread than that of LRP, suggesting that αSYN oligomers may be found throughout the brain earlier in the disease course than can be observed with immunohistochemistry for LRP. Of note, we observed more αSYN oligomers in the hippocampus in patients with cognitive impairment. Given the toxicity of αSYN oligomers, clinicopathological studies focusing on αSYN oligomers may provide insight into PD pathogenesis.

## Supplementary Information

**Additional file 1**. **Fig. S1**. (A) Example of image conversion for stained area measurement by the software ImageJ. (B) Correlation between stained area and neuropil score; **Fig. S2**. Score of αSYN oligomer burden; **Fig. S3** Stained area of αSYN-PLA staining; **Fig. S4** Comparative images of phosphorylated αSYN immunostaining and αSYN-PLA staining.

## Data Availability

The data that support the findings of this study are available from the corresponding author upon reasonable request.

## Abbreviations

αSYN: Alpha-synuclein
H&E: Hematoxylin and eosin
LRP: Lewy-related pathology
NFT: Neurofibrillary tangle
PD: Parkinson's disease
PLA: Proximity ligation assay
RCA: Rolling circle amplification
TBS: Tris-buffered saline
TDP-43: Transactive response DNA-binding protein of 43 kDa
