# Supplementary material for: Discrepancy between distribution of alpha-synuclein oligomers and Lewy-related pathology in Parkinson’s disease
Source: Acta Neuropathol Commun. 2022 Sep 6;10:133. doi: 10.1186/s40478-022-01440-6 (PMC9450240; doi:10.1186/s40478-022-01440-6)
Supplement: Supplementary file 1 — Additional file 1. Fig. S1. (A) Example of image conversion for stained area measurement by the software ImageJ. (B) Correlation between stained area and neuropil score; Fig. S2. Score of αSYN oligomer burden; Fig. S3 Stained area of αSYN-PLA staining; Fig. S4 Comparative images of phosphorylated αSYN immunostaining and αSYN-PLA staining. [file 40478_2022_1440_MOESM1_ESM.pdf]

Supplementary material

Discrepancy Between Distribution of Alpha-synuclein Oligomers and Lewy-Related Pathology in Parkinson's Disease

Hiroaki Sekiya[1,2,3], Asato Tsuji[2,3], Yuki Hashimoto[2,3], Mariko Takata[2,3], Shunsuke Koga[1], Katsuya Nishida[4], Naonobu Futamura[4], Michi Kawamoto[5], Nobuo Kohara[5], Dennis W. Dickson[1], Hisatomo Kowa[2,6], Tatsushi Toda[3,7]

1 Department of Neuroscience, Mayo Clinic, Jacksonville, Florida, USA
2 Division of Neurology, Kobe University Graduate School of Medicine, Kobe, Hyogo, Japan
3 Division of Molecular Brain Science, Kobe University Graduate School of Medicine, Kobe, Hyogo, Japan
4 Department of Neurology, National Hospital Organization Hyogo-Chuo Hospital, Sanda, Hyogo, Japan
5 Department of Neurology, Kobe City Medical Center General Hospital, Kobe, Hyogo, Japan
6 Department of Rehabilitation Science, Kobe University Graduate School of Health Sciences, Kobe, Hyogo, Japan
7 Department of Neurology, Graduate School of Medicine, The University of Tokyo, Bunkyo-ku, Tokyo, Japan

Corresponding authors:

Hiroaki Sekiya, MD, PhD
Address: 4500 San Pablo Road, Jacksonville, FL 32224
Phone: 904-956-8377, Fax: 904-953-7117
Email: sekiya.hiroaki@mayo.edu

Tatsushi Toda, MD, PhD
Address: 7-3-1 Hongo, Bunkyo-ku, Tokyo 113-8655 Japan
Phone: +81-3-5800-6542, Fax: +81-3-5800-6548
Email: toda@m.u-tokyo.ac.jp

(A)

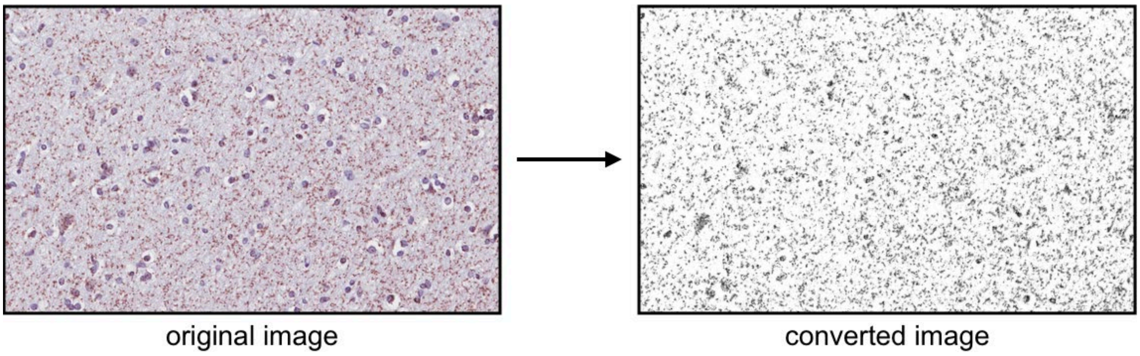

(B)

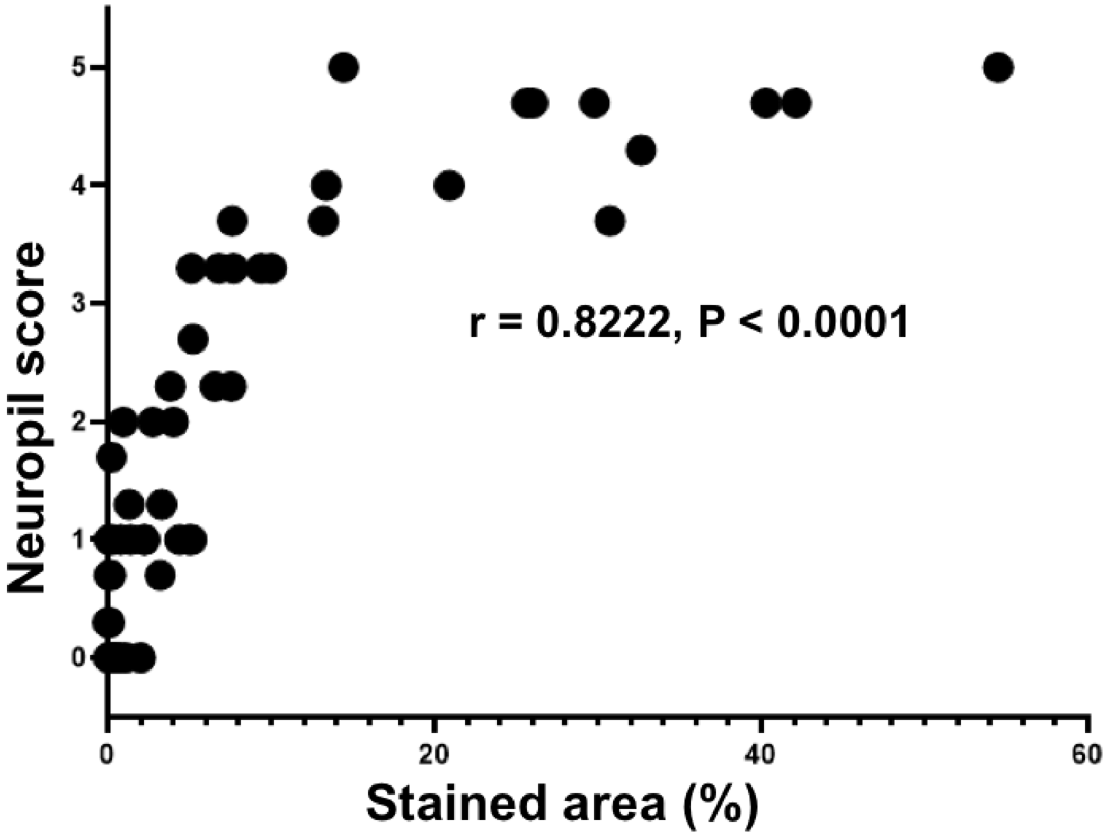

Fig. S1 (A) Example of image conversion for stained area measurement by the software ImageJ (B) Correlation between stained area and neuropil score

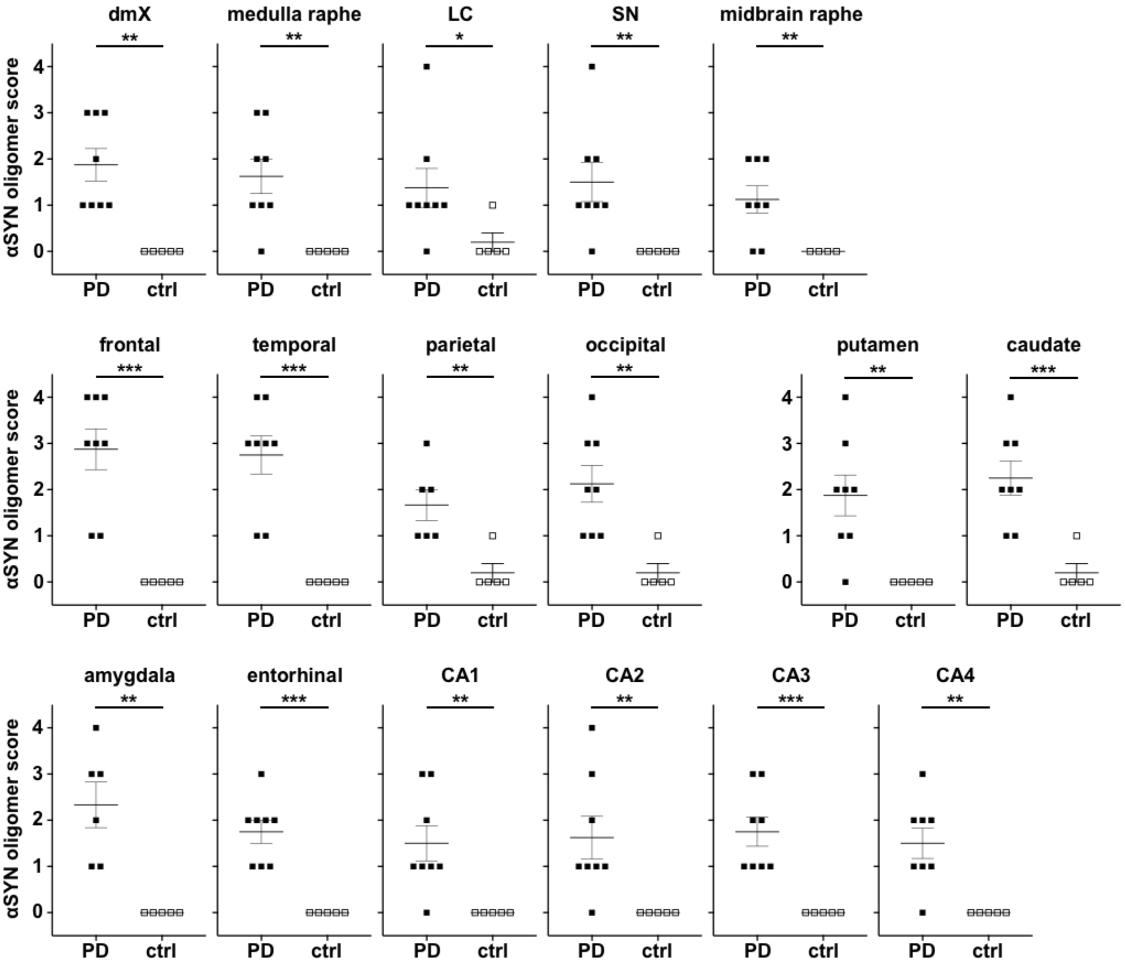

Fig. S2 Scores of α-synuclein oligomer burden

PD, Parkinson's disease; *ctrl*, control subjects; *dmX*, dorsal motor nucleus of the vagus; *LC*, locus coeruleus; *SN*, substantia nigra; *frontal*, frontal cortex; *temporal*, temporal cortex; *parietal*, parietal cortex; *occipital*, occipital cortex; *caudate*, caudate nucleus; *entorhinal*, entorhinal cortex; *CA*, cornu ammonis; * P < 0.05, ** P < 0.01, *** P < 0.001

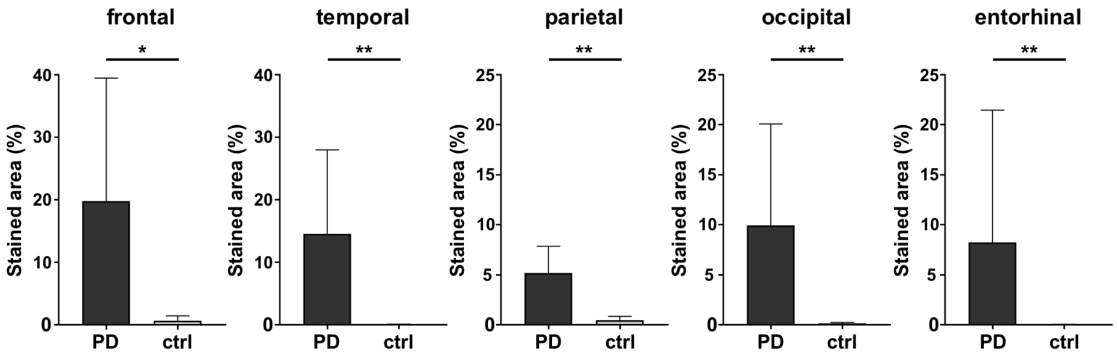

Fig. S3 Stained area of αSYN-PLA staining

PD, Parkinson's disease; *ctrl*, control subjects; *frontal*, frontal cortex; *temporal*, temporal cortex; *parietal*, parietal cortex; *occipital*, occipital cortex; *entorhinal*, entorhinal cortex; * $P < 0.05$, ** $P < 0.01$

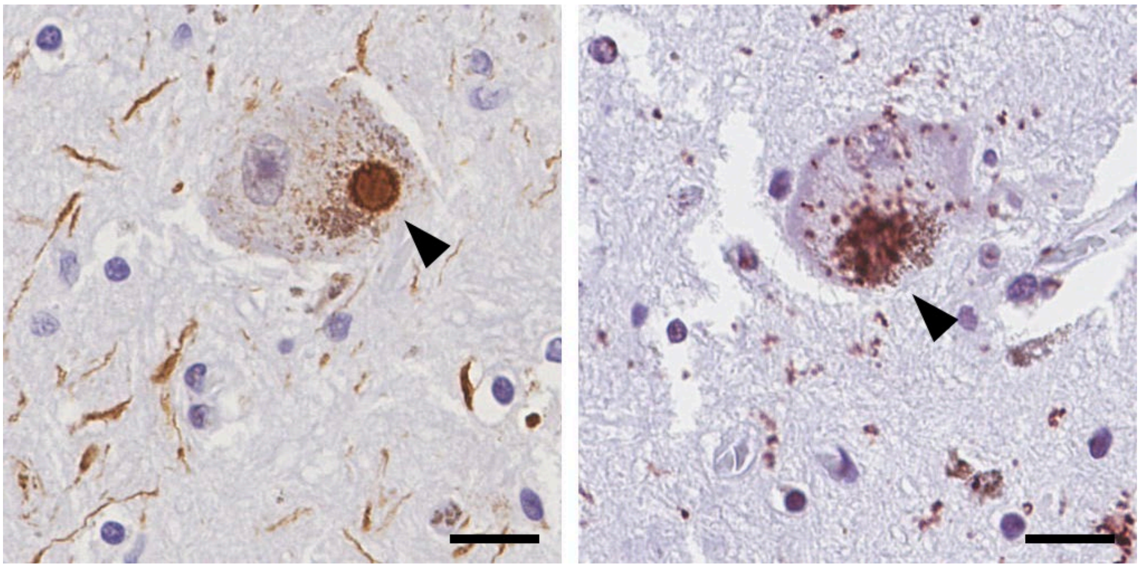

Fig. S4 Comparative images of phosphorylated α-synuclein (αSYN) immunostaining and αSYN proximity ligation assay (αSYN-PLA) staining.

The left image is phosphorylated-αSYN immunostaining and the right image is αSYN-PLA staining. Both images are the substantia nigra in the midbrain. Scale bars 20 μm
